# Supplementary material for: Identification of photoperiod-induced specific miRNAs in the adrenal glands of Sunite sheep (Ovis aries)
Source: Front Vet Sci. 2022 Jul 22;9:888207. doi: 10.3389/fvets.2022.888207 (PMC9354845; doi:10.3389/fvets.2022.888207)
Supplement: Supplementary file 1 [file Data_Sheet_1.ZIP › Supplementary file/miRDeep2 file.zip!/miRDeep2 file/Novel_Structure/SP42A3/Novel_220.pdf]

Provisional ID	:	NC_019467.2_15851
Score total	:	1.6
Score for star read(s)	:	-1.3
Score for read counts	:	0
Score for mfe	:	1.3
Score for randfold	:	1.6
Score for cons. seed	:	
Total read count	:	545
Mature read count	:	545
Loop read count	:	0
Star read count	:	0

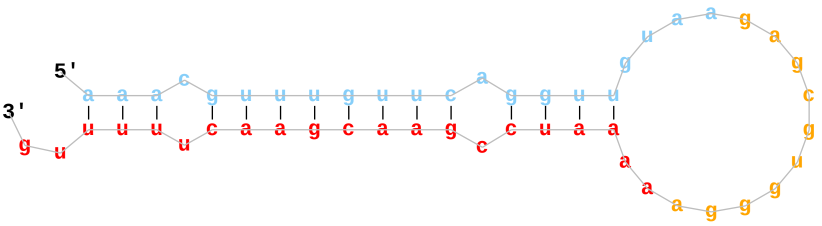

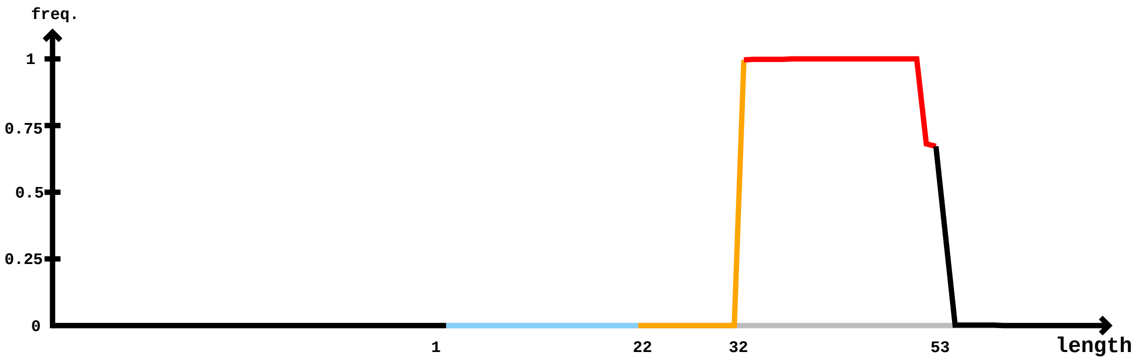

Star Mature

```
5'- uggguucuguccuagguaaugguaguacuggguuggccaaaacguuuguucagguuguaagagcgugggaaaaauccgaacgaacuuuuuggccaagucaauaauuug   -3'    exp
    ..(((.(((.((........))))).)))((.((((((((((.((((((((.((((................)))).)))))))).)))))))))).)).........     reads      mm         sample
    ......................................................................aaaauccgaacgaacuuuu...................     173        0          seq
    ......................................................................aaaauccAaacgaacuuuuu..................     5          1          seq
    ......................................................................aaaauccgaacgaacuuuuug.................     362        0          seq
    ......................................................................aaaauccAaacgaacuuuuug.................     3          1          seq
    ......................................................................aaaauccAaacgaacuuuuugg................     1          1          seq
    .......................................................................aaauccgaacgaacuuuu...................     1          0          seq
    ...........................................................................ccgaacgaacuuuuuggccaag...........     1          0          seq
```
